# Healthy London

**Published:** 1920-07-24

**Authors:** 


### Healthy London.

The modern woman comes in for some hard knocks in these days, but she has nearly always been modern to her contemporaries, and perhaps it is not wise to be too miserable about present tendencies. One of the most frequent exhortations addressed to her nowadays, when she is entering new phases of industry and being admitted to the professions, to the vote and to Parliament, is to remember that her greatest function is motherhood. Judging by recent statistics, she is not allowing these fresh distractions to make her forget, for not only is the record of births rising, but the mortality of infants is decreasing. This means not only more mothers, but better motherhood, and this in a period of considerable stress and difficulty.

These points are well illustrated by the report of the London County Medical Officer (Dr. W. H. Hamer) for 1919. It shows that in the closing weeks of the year the births were in excess of all previous figures, and the increase has been maintained during 1920. Moreover, more marriages took place in London last year than in any previous year, except 1915. There was a substantial reduction in mortality, and the death-rate among infants under a year was the lowest on record.

The yearly average of births during the pre-war period 1911-14 was 111,000, and in the first quarter of this year 36,303 births have been registered. The deaths among the civil population during 1919 are expressed in a rate of 13.6; the corresponding rate in years back to 1916 being 19.2, 15.7, and 14.6. The infantile death-rate was 85 per 1,000; the lowest rate previously recorded was 91, in 1912. One effect of the increased number of marriages has been to aggravate the house shortage.

With the exception of scarlet fever and diphtheria, there was a decline in most of the cases of infectious diseases and of deaths therefrom. Eleven cases of anthrax were notified, four of which were attributable to infection from shaving-brushes manufactured in Japan. Improvement is recorded in the health of school children—a fact noted by medical officers in other parts of the country—and Dr. Hamer considers this improvement good beyond expectation. At the same time, 83,000 children in the elementary school children were found during 1919 to require treatment for one or more defects, but a considerable proportion of these were doubtless of a minor character. Altogether, Dr. Hamer has been able to give a good account of London.

